# Climate adaptation in the southwest US: The SWPar4.5 parameter set for stochastic weather generators

**DOI:** 10.1038/s41597-025-06102-5

**Published:** 2025-11-19

**Authors:** Andrew T. Fullhart, Shang Gao, Wenting Wang, Emile Elias, Gerardo Armendariz, David C. Goodrich

**Affiliations:** 1 School of Natural Resources and the Environment, University of Arizona, Tucson, 85721 USA; 2 Department of Geographic Science, Beijing Normal University Zhuhai Campus, Zhuhai, 519087 China; 3 Southwest Climate Hub, USDA, Las Cruces, 88001 USA; 4 Ecoimpact Solutions, Durango, 81301 USA; 5 Southwest Watershed Research Center, USDA-ARS, Tucson, 85719 USA

**Keywords:** Environmental health, Atmospheric dynamics

## Abstract

Climate assessment in the southwestern US is complicated by extreme spatial gradients and short, intense rainfall events. In this region, gridded climate data, including commonly applied ~4 km daily datasets, often obscure spatial gradients and short-term precipitation dynamics. Contrasting, point-scale data (as measured by ground instruments like rain gauges) better reflects important precipitation factors and is preferred input for certain site-specific environmental models, including models classified by their domain as point-, plot-, field-, and hillslope-scale models. Facilitating targeted climate assessment, Southwest Parameter Set 4.5 (SWPar4.5) enables a framework for creating probable historical and future point-scale climate time series at ~800 m resolution. SWPar4.5 provides monthly climate benchmarks to parameterize a stochastic weather generator estimated using a data fusion of two existing gridded climate projections with differing spatiotemporal resolutions based on the middle ground representative concentration pathway scenario (RCP4.5). Resulting daily time series contain basic weather variables and include sub-daily precipitation patterns. Increases in precipitation intensity at local scale were found, with implications for soil erosion, runoff, and other environmental indicators.

## Background & Summary

When used as input for site-specific, small-scale hydrologic and climatological assessment, different gridded climate datasets can yield a range of outcomes for key results^1–3^. The spatiotemporal data processing done to produce the various resolutions of gridded climate data may lead to information related to local dynamics becoming indistinct or lost. This particularly applies to precipitation variables, which are commonly subject to downscaling methods. For gridded precipitation data, accumulation is typically represented as an areal average, i.e., accumulation is spatially distributed across the spatial extent of a grid cell, often resulting in small daily accumulation amounts and misrepresentation of dynamics that may occur at finer scales than the resolution of gridded climate data, such as convective rainfall. The term “drizzle effect” has been used to describe the tendency of coarse precipitation products to have both reduced intensity and a greater number of time steps with precipitation occurrence than what is observed by a ground station^4,5^. Therefore, when considering site-specific hydrometeorological factors, the incompatibility between the small spatial scale of an area of interest and coarser scales of gridded data has the potential to result in bias. Spatial climate gradients and characteristic storm patterns are further examples of misrepresented properties in coarse climate data, though these are important when assessing localized changes in hydrometeorological and climatological norms.

Issues related to the scale of gridded climate data are particularly obvious in the diverse climatology of the southwestern US, defined here as a ~1.1 million km^2^ area comprising the states of Nevada, Utah, Arizona, and New Mexico. This mountainous coverage area largely overlays the physiographic regions referred to as the Desert Southwest, the Great Basin, and the Colorado Plateau. The rangeland and desert environments in the generally hot and dry valley basins are characterized by punctuated rainfall events and significant interannual variability, while numerous high elevation mountain ranges have climates that contrast drastically, with greater precipitation, colder temperatures, and snowfall being common^1,6,7^. Steep elevational gradients that are typical in the topography of the Basin and Range Province result in climate gradients in coarse climate images being represented by a minimal number of grid cells. The characteristic features that are present highlight the need to achieve greater spatiotemporal resolutions for climate datasets in the western United States^1,3^.

It is becoming more common practice to consider climate projections in environmental assessments to identify adaptation and mitigation measures, use adaptive management and resilience planning, and understand how projected changes may interact with management decisions^8–10^. Bias related to the coarseness of gridded climate data has the potential to affect environmental assessments done in the southwestern US, which may include rangeland health assessments, wildfire threat management, ecological inventory, and others. For some places in the region, environmental indicators have already shown adverse responses to climate change, such as increases in bare ground percentage, increased soil loss, changes in ecological state due to invasive plant spread, and decreased water yield^11–14^. These are factors monitored by local, state, and federal resource management agencies, private land managers, and others. In particular, federal agencies are responsible for managing a significant proportion of land in the study area, with an average of approximately 53% of land area being federally managed for the states covered by SWPar4.5^15^. Economic damages from weather-related disasters totaled approximately 67 billion USD between 2018 and 2022 in the broader southwestern US, reflecting social impacts of extreme weather which is expected to be exacerbated by climatological change^16^. Trends existing in SWPar4.5, particularly for temperature and factors tied to precipitation intensity, have implications for runoff and erosion rates, and are relevant to rangeland processes. In fact, several rangeland health indicators identified in technical guides may be assessed using the historical and future climate data in SWPar4.5^17^.

### The southwest parameter Set 4.5 (SWPar4.5)

To enable greater scope of climate assessment, the Southwest Parameter Set 4.5 (SWPar4.5) presented here provides parameter sets needed by stochastic weather generators (SWGs). SWGs are statistical climate models that generate non-deterministic synthetic time series, often at daily temporal resolution, which serve the needs of numerous applications and require less computational expense than dynamical downscaling^18^. Such computational efficiency enables the generation of long-term time series that better capture the random nature of weather systems. Guidelines from the Intergovernmental Panel on Climate Change (IPCC) recognize the use of SWGs as a strategy for impact assessment and characterization of baseline climates^19^. Moreover, SWGs and stochastic disaggregation techniques have been used to downscale future projections from global climate model (GCM) outputs, including those involved in the IPCC climate scenarios represented in the Coupled Model Intercomparison Project (CMIP) and accompanying series of GCM ensembles^18,20^. Typically, point-scale SWGs are meant to represent site-specific conditions and localized precipitation dynamics. Therefore, they are useful for providing approximations of ground records and reproducing key statistical factors present in long-term records. Stochastic time series can also be used in the context of risk assessment for extreme events because they can be made arbitrarily long, allowing them to extend ground records and effectively produce a time series with statistical distributions representing the full range of weather events, including the rare, extreme events (usually within a stationary climate).

SWGs often operate by temporally disaggregating monthly climate benchmarks that represent statistical reductions of daily and/or sub-daily time series^18,19^. These input parameters must always be derived for long-term windows in order to be representative of climate. Therefore, to represent climate change, the SWPar4.5 dataset consists of separate parameter sets that are estimated for a series of eight 30-year windows within the 2000–2099 period at decadal intervals and one historical window representing the 1974–2013 period. For these windows, critical parameter values were adjusted relative to historical baselines to reflect climate change according to the “middle ground” emissions scenario (in this case, the RCP4.5 scenario in the CMIP5 generation, discussed later). For each time window, an SWG may be applied to generate a point-scale climate record within a stationary climate regime (no climate signal). The resulting data is non-deterministic and may lack the cycling present in GCMs, such as the El Niño-Southern Oscillation (ENSO) and intra-decadal and decadal-scale cycles of drought and pluvial. However, the resulting ~800 m resolution of daily and sub-daily information represented by SWPar4.5 is at higher resolution than currently given by most climate projections. Consequently, this enables a number of long-term averages to be represented at high resolution, some of which cannot be calculated from the original climate projections. This higher resolution is especially needed where there is complex terrain and strong spatial gradients. Furthermore, the dataset generally allows climate inputs to be obtained for a number of site-specific environmental assessment applications.

SWPar4.5 used an efficient framework that minimized the required number of steps and assumptions for production of gridded SWG parameter maps representing various climate variables. One benefit of the framework is that pre-existing data sources are selected and applied as directly as possible, such that major factors like monthly average accumulation and temperature are taken exactly from existing gridded datasets. As such, the approach may make use of a range of available datasets, and depending on the dataset and variable, known uncertainties from the original dataset may be transferred to corresponding SWGs. In the case of SWPar4.5, the parameter sets could be created at ~800 m resolution (30 arc seconds) by relying on a high resolution monthly-scale climate dataset to obtain spatial information on primary climate gradients. Additionally, for certain precipitation parameters, a coarser daily-scale dataset (1/24 arc degree) with the same selection of climate scenarios and GCMs was used to inform the statistical downscaling approach with equivalent grid-scale parameters that allow the difference between grid- and point-scale values to be quantified (for the historical period). Multiple statistical downscaling techniques were also warranted due to the variety of weather variables being represented. The estimation of point-scale precipitation parameters principally involved the following techniques: empirical Bayesian Kriging, gradient boosting, and delta downscaling. As will be outlined in the methodology, these techniques each played a role in the production of parameter maps in SWPar4.5. For other required parameters of temperature and solar radiation, it was considered sufficient to approximate their point-scale parameter values in SWPar4.5 via direct calculation from high resolution gridded data. In general, the framework may be similarly applied to other sources of gridded climate projections with additional consideration of their own quality controls and uncertainties, as well as their own strengths and weaknesses. This may entail applying the framework in other regions where available data may have lesser resolutions or less dense ground networks. In these cases, it is possible that similar prediction performance may be attained as in the present work given that this coverage area represents an especially challenging area for climate prediction and more uniform climate gradients are common elsewhere.

Furthermore, tools were created to query the SWPar4.5 parameter set: a website allows a single parameter set to be downloaded for a selected time and place, and secondly, a command-line tool allows batch queries. The text formatting of the parameter set files that are produced enables SWPar4.5 to be readily integrated with a widely applied SWG called CLIGEN^21^. CLIGEN uses monthly average parameter sets (given by SWPar4.5) and a Richardson-type framework for daily sequencing of wet/dry days based on Markov chain transition probabilities. Skewed normal distributions are used for non-zero single-day accumulation amounts and daily maximum and minimum temperatures^21^. There is notable overlap in required parameter sets between CLIGEN and other daily SWGs. However, CLIGEN additionally requires two sub-daily parameters that are involved in generating idealized stochastic storm hyetographs shaped by a single peak: monthly maximum 30-minute intensity values and normalized time-to-peak intensity probability distributions (the latter parameter is a further exception because it is not monthly-scale). There are several computer models in particular dealing with agricultural, environmental, and hydrological applications that rely heavily on CLIGEN or other SWGs, though generated CLIGEN outputs may be adapted to many other models. For example, CLIGEN is directly integrated with the Water Erosion Prediction Project (WEPP) model for agricultural settings and the Rangeland Hydrology and Erosion Model (RHEM) for rangeland management^22,23^. Erosion simulations particularly benefit from CLIGEN’s realistic daily rainfall frequency distributions and the fact that its sub-daily precipitation information is congruent with the time scales over which runoff generation occurs that in turn drives water erosion.

As described in Table 1, the methodology used to produce SWPar4.5 relies on the availability of existing downscaled gridded climate projections, as well as a dense ground network of parameter sets to establish historical baselines of point-scale parameter values. There are a number of existing data products with coverage of the U.S. that have downscaled projections based on the outputs of various greenhouse gas emission scenarios and GCMs. Generally, there is a tendency for monthly-scale climate datasets to have the highest available spatial resolutions. Examples of monthly datasets covering the U.S. with a relatively high resolution of 1/120° (30 arc seconds or ~925 m at the equator) include PRISM Monthly^24^, WorldClim^25^, and NEX-DCP30^26^. In comparison, a common resolution for daily-scale climate datasets is 1/24° (~4 km at the equator), which includes PRISM Daily^24^, GridMet^27^, and MACA^28^ (the 1 km DayMet dataset^29^ is additionally compared herein). Two of these datasets, NEX-DCP30 and MACA, represent simulated climate projections that include both historical and future periods, and were used in the production of SWPar4.5. Closed climate simulations given by GCMs may not result in the same accuracy of baselines as observation-based datasets that represent only historical baselines, such as ground datasets or datasets that use observations to refine the accuracy of a record given by a predictive model (e.g. PRISM). However, reasonable agreement was found for critical baselines in the applied GCMs. As done in the present work, information from GCM simulations could be blended with observation-based data by consideration of only long-term averages, which may be expected to be equally represented by both data types, unlike in short-term comparisons, where differences may be expected. Also important for SWPar4.5 was the availability of observed parameter sets in the region from the U.S. CLIGEN ground network representing a 40-year period^30^. In the present framework, these parameter sets were treated as target values for predictive models that were used to map a set of historical baseline parameters. In the precipitation downscaling approach, these historical maps provided initial values for subsequent parameter adjustments reflecting climate change.

**Table 1:** Datasets involved in the production of SWPar4.5 and compared sources of climate data.

Data Sources Information				
Southwest Parameter Set 4.5 (SWPar4.5)				
SWPar4.5	30 arc sec. (~800 m)	Monthly	1974–2099	Present dataset
Queried records used for production of SWPar4.5				
NEX-DCP30	30 arc sec. (~800 m)	Monthly	1974–2099	Thrasher *et al*.^26^
MACAv2	1/24 arc deg. (~4 km)	Daily	1974–2099	Abatzoglou and Brown (2012)^28^
U.S. CLIGEN Net.	Point-scale	Parameter Set	1974–2013	Srivastava *et al*.^30^
3DEP 10-Meter DEM	~800 m (resampled)	—	—	U.S. Geological Survey^45^
Queried records used for comparison to SWPar4.5				
DayMet	1 km	Daily	1980–2014	Thornton *et al*.^29^
GridMet	1/24 arc deg. (~4 km)	Daily	1979–2014	Abatzoglou (2012)^27^
PRISM	1/24 arc deg. (~4 km)	Daily	1981–2014	Daly *et al*.^24^
GHCNd	Point-scale	Daily	1974–2013	Matthew *et al*.^52^

An intermediate emissions scenario often referred to as the “middle ground” scenario is represented in SWPar4.5 called RCP4.5. This scenario is defined by the IPCC as having an additional 4.5 W/m^2^ of radiative forcing by the year 2100 compared to a historical baseline^31^. In order to capture shifts in climate zones in this scenario, which may be localized and topographically controlled, the highest available spatial resolution data is needed. To this end, the SWPar4.5 dataset is derived from downscaled CMIP products from a previous CMIP generation (CMIP5) because of greater availability of highly downscaled products for CMIP5 relative to CMIP6. In the two existing gridded climate projections used herein, NEX-DCP30 and MACA, the CMIP5 ensemble was downscaled to differing spatiotemporal resolutions, with both datasets including 21 common GCMs from the larger CMIP5 ensemble. In a data blending approach, average monthly statistics (e.g., accumulation and mean temperatures) were taken exactly from the monthly-scale NEX-DCP30 at ~800 m resolution, while daily-scale statistics were resampled to this resolution from the coarser daily-scale MACA projections and were used to adjust baseline values for future time windows, or were used to directly calculate parameters MACA where possible.

In the present framework, the sub-set of models from the CMIP5 ensemble was used to derive regional ensemble statistics by spatially averaging NEX-DCP30 for the study area. Based on this, selection of three GCMs with contrasting precipitation outcomes were chosen to be represented in SWPar4.5 that results in three series of parameter sets in the overall dataset. A regional ensemble trend showed that the increase in daily maximum temperature is 2.7°C by 2100 in this scenario, similar to the global average^31^, though the ensemble average precipitation suggests the southwestern U.S. will see relatively small change in long-term average annual precipitation. Nonetheless, important spatial and temporal changes in precipitation were predicted in SWPar4.5, and notably, precipitation intensity metrics were shown to increase despite annual precipitation remaining close to historical averages. It is important to note that some periods in the RCP4.5 projection have fast rates of change, particularly in the first half of the century, and the discrete intervals used in SWPar4.5 represented by 30-year windows may not adequately capture these because climate stationarity is assumed in each time window. Although climate parameters are updated to represent non-stationarity in the dataset as a whole, SWGs that explicitly allow non-stationary through use of time-dependent input parameters may be better suited to reflecting short-term dynamics in certain periods of the projection.

**Fig. 1:**
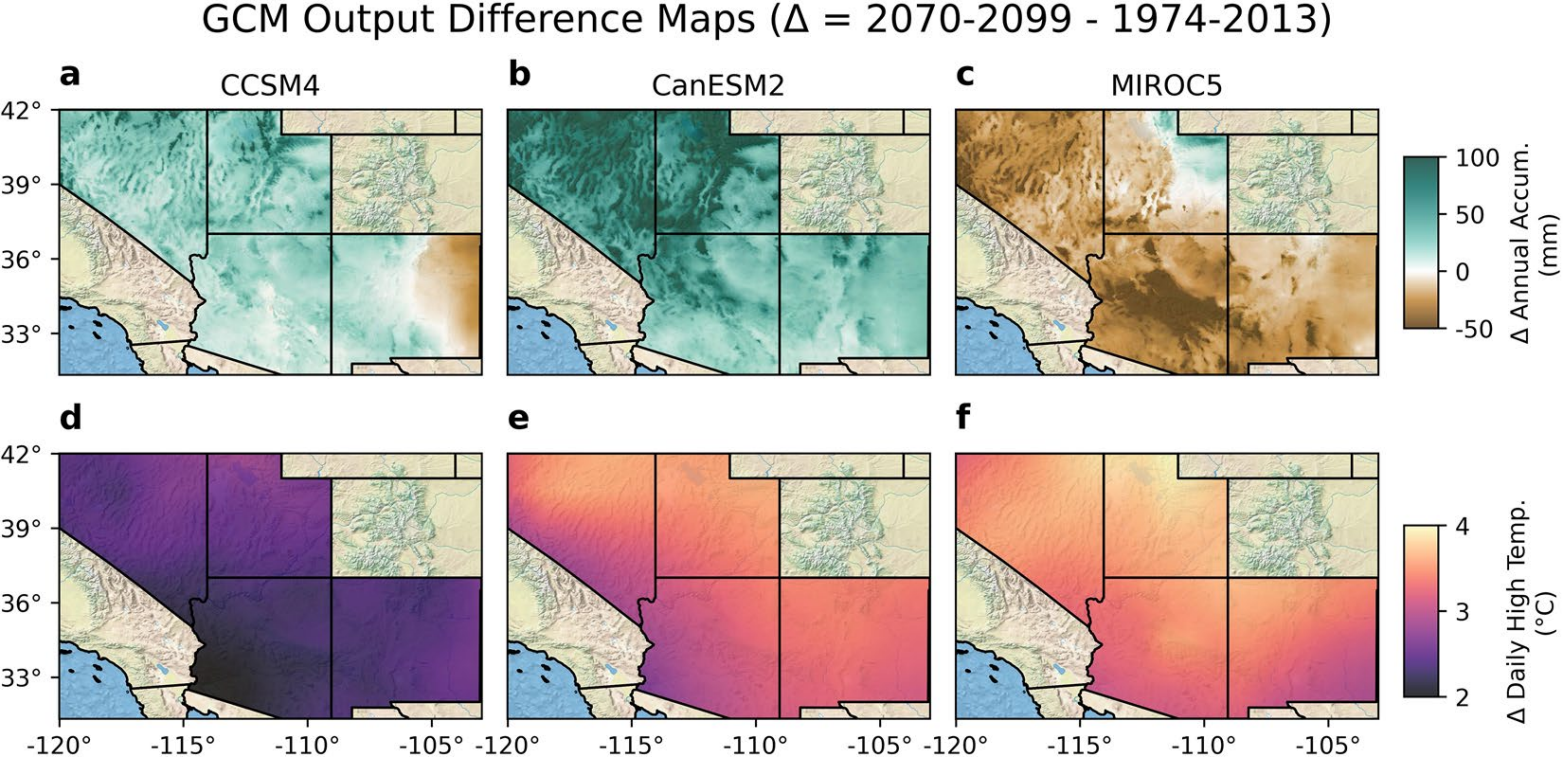
GCM spatial changes in long-term averages for (**a**) CCSM4 accumulation difference, (**b**) CanESM2 accumulation difference, (**c**) MIROC5 accumulation difference, (**d**) CCSM4 temperature difference, (**e**) CanESM2 temperature difference, and (**f**) MIROC5 temperature difference. Nine parameter sets were developed for each GCM corresponding to time intervals that include those seen here. These primary spatial gradients were calculated directly from the NEX-DCP30 dataset.

The corresponding spatial variations in climate change for each GCM are shown in Fig. 1 in terms of mean accumulation and temperature with important differences being visually evident. Figure 1 demonstrates the fact that a range of outcomes is yielded by the GCMs represented in SWPar4.5. The CCSM4 model is representative of the regional ensemble mean of annual precipitation, while CanESM2 has relatively high annual precipitation, and MIROC5 has relatively low annual precipitation. In terms of spatial variation, the three models are in closer agreement for temperature outcomes than precipitation outcomes. Figure 1 also illustrates the fact that site-specific GCM trends may differ from those that would be expected given regional GCM trends. Use of a stochastic weather generator affords the ability to readily generate different realizations of time series using one or more parameter sets, allowing differences between GCMs to be understood and for uncertainties to be derived related to variability occurring within one or more records. Depending on the application for SWPar4.5, it may therefore be possible to determine site-specific uncertainties based on the available data for a given site. For example, sources of uncertainty may include the effect of strong inter-annual variability on any determined long-term average, which may be quantified using multiple time series realizations.

### Precipitation factors in SWPar4.5

**Fig. 2:**
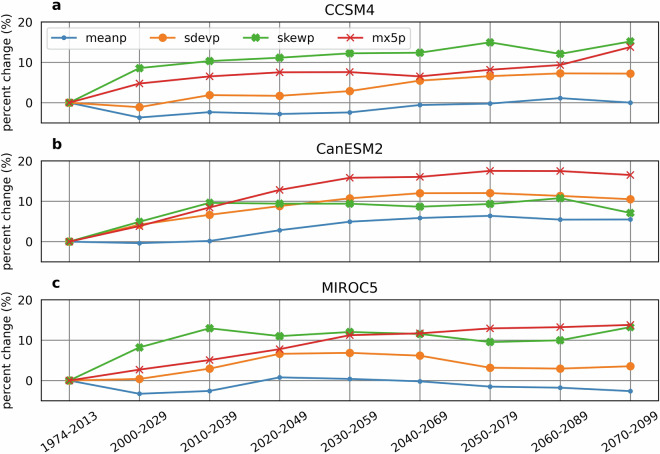
Regional trends for four CLIGEN precipitation parameters in SWPar4.5 for the three selected GCMs and nine long-term time windows for (**a**) CCSM4, (**b**) CanESM2, and (**c**) MIROC5. To show a single trend for all monthly parameters, the parameters were spatially averaged, as well as monthly averaged using the average number of days in the month with precipitation as a weighting factor. Rates of change in the MEANP, SDEVP, and SKEWP variables of SWPar4.5 are derived from the MACA-based surfaces, while point-scale parameter values in the 1974–2013 window were used as the starting point. Rates of change in the MX.5 P variable were derived from gradient boosting regression model predictions.

Figure 2 shows regionally averaged precipitation parameter trends present in SWPar4.5. These include the monthly mean (MEANP), standard deviation (SDEVP), and skewness (SKEWP) of non-zero daily accumulation (in this case, the monthly parameters are weighted by the number of wet days per month to allow single trends to be plotted). Also shown is the monthly mean 30-minute maximum intensity (MX.5 P). The parameters have a tendency of trending upwards, suggesting a general increase in regional precipitation intensity. Increased precipitation intensity is expected, based in part on relationships between air temperature and precipitation. From the Clausius-Clapeyron equation, which describes the rate of change of saturated vapor pressure and moisture holding capacity of air for a given change in air temperature, it follows that maximum precipitation intensity will correspondingly change at a rate referred to as C-C scaling (~7% per °C)^32^. As warmer air has higher saturated vapor pressure, it may be expected that the most extreme rainfall events, which occur when air is close to saturation, are likely to increase in intensity. While it cannot be said that extreme rainfall is expected to occur in all calendar months, a monthly CLIGEN parameter closely tied to extreme rainfall intensity is MX.5 P. From the plot, it may be seen that averaged MX.5 P increases by roughly 10–16% for the 2070–2099 period depending on the GCM, which is close to the increase of 19% expected by C-C scaling for the 2.7°C regional temperature increase. Furthermore, trends in the three CLIGEN precipitation parameters that characterize the statistical distribution of daily accumulation trends are also suggestive of increases in intensity. MEANP is the most obvious indicator of daily intensity of these parameters, though MEANP doesn’t show substantial change in SWPar4.5 compared to SDEVP and SKEWP, which both increase at greater rates. This may be in disagreement with observed rates of change in MEANP and SDEVP for the region^33^, which have shown SDEVP increasing at a rate similar to that given in SWPar4.5, while MEANP was observed to likewise increase at a similar rate. Therefore, MEANP may require further investigation as a possible source of error and critical factor for shaping the daily rainfall distribution. At the same time, the parameter trends of SWPar4.5 are generally consistent with global observations that precipitation is becoming increasingly “uneven”, whereby precipitation distributions are becoming more skewed towards larger events, and greater fractions of total precipitation are occurring on the wettest days of the record^34^. Corresponding to these observations, unevenness in SWPar4.5 is increased by the upward trends in both SDEVP and SKEWP. Given the fact that regional total accumulation is relatively stable in the projections, while point-scale precipitation factors suggest important changes towards greater intensity, it is apparent that much of the change in precipitation is temperature driven. Temperature driven change is obvious when considering factors such as snow accumulation, though less obvious factors like the relationship of precipitation intensity with temperature may be reflected in the CLIGEN parameter sets and the generated time series.

**Fig. 3:**
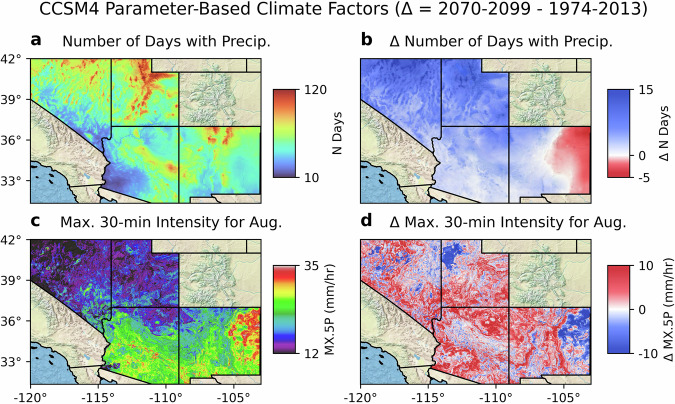
Parameter-derived climate factor maps from the CCSM4 parameterization in SWPar4.5 including (**a**) annual number of days with precipitation occurrence, (**b**) change in annual number of days with precipitation occurrence, (**c**) MX.5 P for August, and (**d**) change in MX.5 P for August. The number of days with precipitation occurrence may be calculated from the parameter set (as well as from time series output), while MX.5 P directly represents predicted parameter surfaces from gradient boosting regression.

**Fig. 4:**
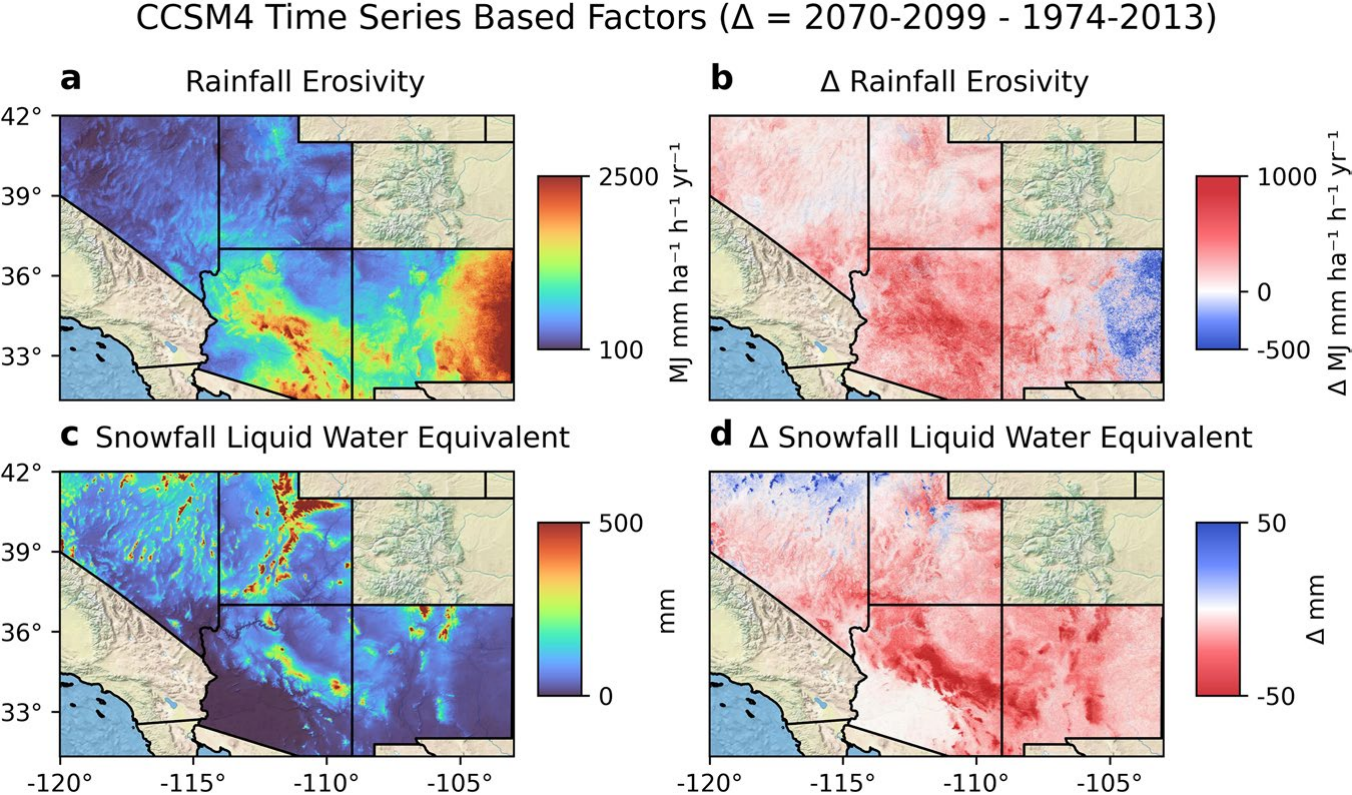
Climate factor maps showing examples of various possible climate factors derived from CLIGEN time series parameterized by CCSM4 including (**a**) Annual rainfall erosivity, (**b**) change in annual rainfall erosivity, (**c**) annual liquid water equivalent of snowfall, and (**d**) change in annual liquid water equivalent of snowfall. An analytical solution for calculation of annual rainfall erosivity was used that considers the sub-daily precipitation pattern given by CLIGEN. Snowfall was calculated as precipitation occurring on days where the midpoint temperature between the daily maximum and minimum was <5 °C.

A major advantage of SWPar4.5 is the ability to calculate a wide variety of climate factors with a geospatial perspective. These factors may be calculated from the resulting daily-scale weather data and sub-daily precipitation hyetographs, as well as from parameters values themselves. To illustrate the range of metrics that can be calculated, some of which cannot be calculated from other datasets, examples of parameter-based factors are shown in Fig. 3 and examples of factors derived from output time series in Fig. 4. Deriving factors from CLIGEN output time series for large gridded parameterizations like SWPar4.5 has higher computational demand and was performed on a cluster computer for the present example. The coverage area of SWPar4.5 consists of approximately 1.6 million parameter sets for a given time window (and as many separate CLIGEN simulations). When based on time series, climate factors may be easily separated for a time frame of interest, such as for the growing season, monsoon season, wet/dry seasons, etc., which cannot necessarily be done with the original high-resolution monthly-scale data or SWG parameter sets themselves. While examples of factors are varied, they may include record extremes, intermittency, sub-daily precipitation dynamics, inter- and intra-annual variability, etc. Warranting further investigation, the blending of data inputs from the original gridded projections and the stochastic temporal disaggregation done by CLIGEN allows for consideration of factors over a variety of time frames. The dataset may display changes for short-term time windows at monthly and seasonal scales related to, e.g., growing season and monsoonal dynamics, with the shorter time frames being sufficient in temporal resolution to enable analysis of convective rainfall dynamics. For a more comprehensive assessment of CLIGEN output, previous works like Mehan *et al*.^35^ and Wang *et al*.^36^ have validated a number of sub-daily and daily precipitation factors (including extreme precipitation) using time series generated from ground-based parameter sets so that parameter error can be considered negligible.

In Fig. 3, two parameter-based factors are shown, being the annual number of days with occurrence of precipitation (i.e., the number of “wet” days), and the maximum 30-minute intensity (MX.5 P) for the month of August, which is during the North American monsoon season. Both factors are generally suggestive of increases in intensity. There is a tendency for the number of wet days to increase in correspondence to the increases in precipitation predicted for a large proportion of the coverage area by CCSM4. In Fig. 4, two factors derived from time series are shown. These are the rainfall erosivity index and the liquid equivalent of snowfall. The rainfall erosivity index reflects the climatic potential for soil erosion caused by rainfall and runoff, or more simply, the vigor of rainfall^37^. Originally, it was used as a parameter in the Universal Soil Loss Equation (USLE), and is defined as the multiplication of two factors calculated for a single rain storm: the kinetic energy of rainfall acting on a unit area and the maximum 30-minute intensity. Rainfall erosivity increases when accumulation and/or intensity are increased, and therefore, the calculated rainfall erosivity factor is sensitive to the precipitation hyetographs generated by CLIGEN. Rainfall erosivity was calculated using RUSLE2^37^ guidelines (R. indicates the revised USLE) and the analytical solution given by Yu (2002)^38^ was used for calculation of necessary components from CLIGEN time series. Both factors in Fig. 4 used a liquid/soil phase threshold for precipitation of 5°C for the daily average temperature, assumed to be equal to the mean of daily maximum and minimum temperatures. This warmer threshold was used with the priority to omit snowfall for estimation of rainfall erosivity with the assumption that it is unlikely that an erosive rain event would occur in the temperature range near this threshold, and that it is more likely that a large snow event would be incorrectly counted as an erosive rain event. In this case, the predicted increases in rainfall erosivity are caused by increased intensities and are compounded by conversion of snow to the liquid phase as a result of temperature increases. Note that for certain factors calculated from CLIGEN time series that are sensitive to extreme values, such as rainfall erosivity, or extreme precipitation frequency itself, spatial auto-correlation may be lower than expected for neighboring grid cells due to the stochastic nature of the time series. This issue is visible in parts of the rainfall erosivity map. Additional post-operations may be needed in similar frameworks, such as to smooth predicted surfaces and perform error analysis on the resulting surfaces.

As shown by the SWPar4.5 dataset, high resolution parameterization of SWGs offers a framework for site-specific assessments that can be based on pre-existing grid-scale datasets. The dataset helps to identify areas expected to experience large degrees of change. It also highlights the fact that, while regional-scale climate change may not appear to be dramatic in some cases, there can be significant localized changes. These spatial changes may vary from one GCM to another, making it important to consider multiple GCMs to better quantify a mean outcome. There may also be significant differences between GCMs in terms of temporal variability occurring on seasonal, decadal, and other time scales. The broad range of outcomes given by SWPar4.5 allow best practices to be followed for incorporating climate projections into land management, which are identified in technical guides developed by the U.S. Department of the Interior and other institutions^39,40^.

## Methods

**Fig. 5:**
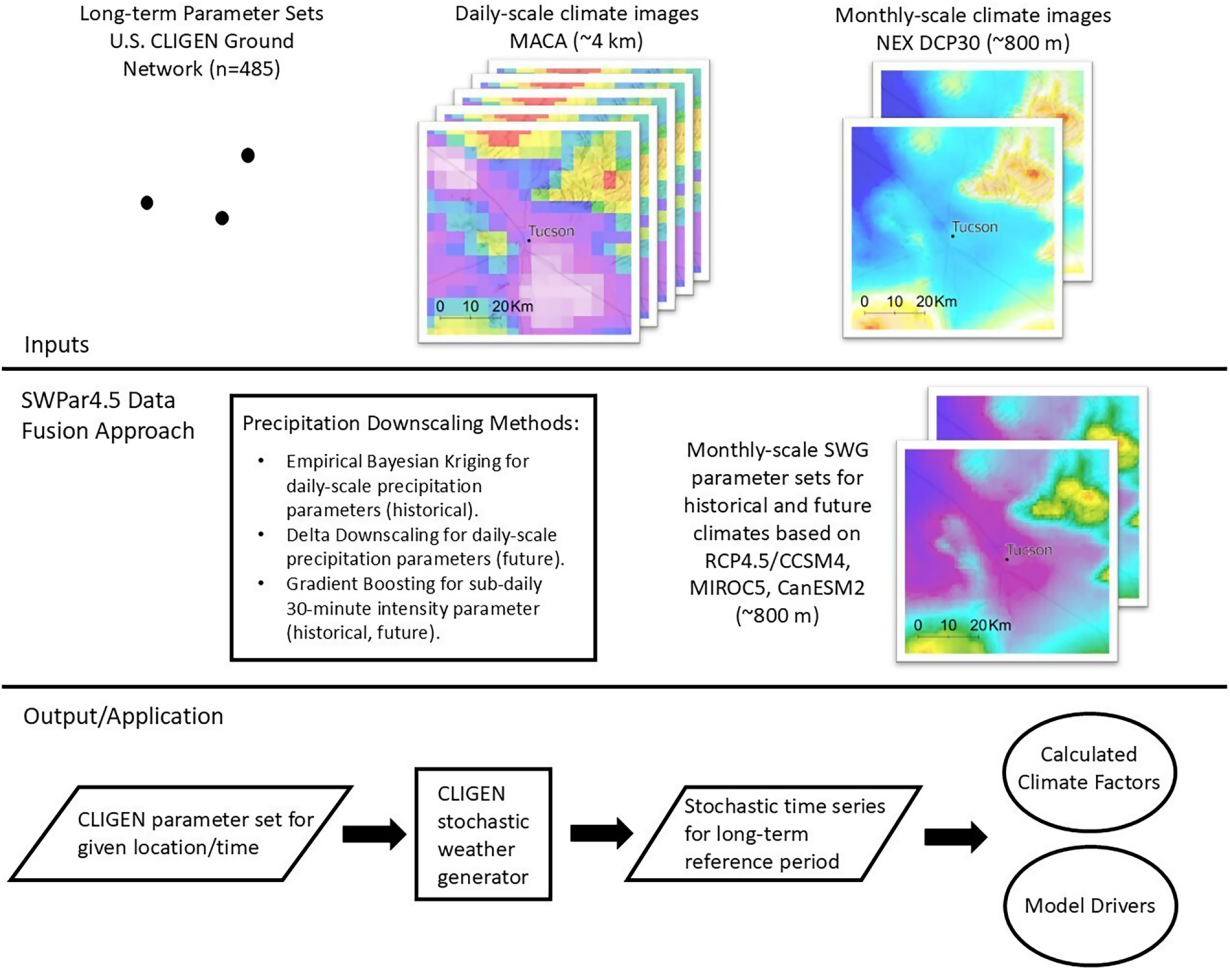
Outline of the SWPar4.5 methodology according to the data inputs, data fusion, and output/application components.

The methodology behind the SWPar4.5 parameter set for the southwest U.S. makes use of several approaches for both estimation and direct calculation of parameters, depending on the parameter. This was warranted because of the variety of data requirements needed to populate site-specific parameter sets. The precipitation downscaling methodology illustrated in Fig. 5 consists of a fusion of two gridded climate datasets with differing spatiotemporal resolutions, as well as ground-based SWG parameter sets. Other climate variables besides precipitation were handled more simply, e.g., temperature and solar radiation parameters, which were directly calculated from high resolution gridded data. However, the extensive parameter set for wind parameters used by CLIGEN is not provided by SWPar4.5, and in order to provide complete parameter sets, wind parameters may optionally be taken from the nearest ground station using the command-line tool described in the Usage Notes section. Taking wind parameters from the nearest station was considered justified because most potential applications for the dataset are either insensitive to wind inputs or do not consider wind at all, but applications that do consider wind should take this into account.

All parameters provided by SWPar4.5 were derived from 30-year windows at decadal intervals beginning with 2000–2029 and ending with 2070–2099, except for the historical window of 40 years from 1974–2013, resulting in nine total parameter sets per GCM. The historical period is defined here as the reference period of the ground network parameters rather than being pre-2006 before the prospective simulation begins in CMIP5. It is possible that errors are higher during the overlapping years with prospective data, but this is likely a small effect given the longer 40-year record length that is used. Three parameterized GCMs were CCSM4, CanESM2, and MIROC5 to represent middle, high, and low-end regional precipitation outcomes, respectively. All calculated CLIGEN parameters and the major methodological components used to determine each parameter are shown in Table 2. Two major software tools were used in this work: Google Earth Engine^41^ to access climate datasets, and ArcPro 3.4^42^ for production of parameter maps the predictive models that were used.

**Table 2:** CLIGEN Parameter information and supporting variables that are mapped in SWPar4.5.

Monthly parameters and supporting variables	Definition	Unit of Measurement	Source Dataset	Notes
ACCUM	Precipitation accumulation.	inches	NEXDCP30	Directly calculated. Involved in wet day count parameter adjustment scheme. Supporting variable.
MEANP	Mean of daily non-zero accumulations.	inches	MACA	Delta downscaling. Involved in wet day count parameter adjustment scheme.
SDEVP	Standard deviation of daily non-zero accumulations.	inches	MACA	Delta downscaling.
SKEWP	Skewness of daily non-zero accumulations.	[−/−]	MACA	Delta downscaling.
P(W|W)	Empirical probability of a wet day following a wet day.	[−/−]	—	Involved in wet day count parameter adjustment scheme.
P(W|D)	Empirical probability of a wet day following a dry day.	[−/−]	—	Involved in wet day count parameter adjustment scheme.
RATIO	Ratio of P(W|W) to P(W|D)	[−/−]	—	Involved in wet day count parameter adjustment scheme.
MX.5 P	Mean of monthly maximum 30-min. intensity	Inches/hr	U.S. CLIGEN Net.	Gradient boosting for all time windows.
TimePk	Empirical CDF datapoints of time-to-peak intensity for all months combined	[−/−]	U.S. CLIGEN Net.	Historical interpolations applied to all time windows. Not a monthly parameter.
Tmax	Mean daily maximum temperature.	°F	NEXDCP30	Directly calculated.
Tmin	Minimum daily maximum temperature.	°F	NEXDCP30	Directly calculated.
SDTmax	Standard deviation of daily maximum temperature.	°F	MACA	Directly calculated.
SDTmin	Standard deviation of daily minimum temperature.	°F	MACA	Directly calculated.
DewPt	Monthly mean of daily average dewpoint temperature.	°F	MACA	Converted from relative humidity.
SolRad	Monthly mean of daily incoming solar radiation.	Langleys/d	MACA	Directly calculated.
SDSol	Standard deviation of daily incoming solar radiation.	Langleys/d	MACA	Directly calculated.
All Wind Pars	Various	—	U.S. CLIGEN Net.	Can be taken from nearest U.S. CLIGEN Net. station using command line tool.

As most of CLIGEN’s precipitation parameters are statistical reductions of daily-scale data, the daily-scale MACA dataset was more involved than the monthly-scale NEX-DCP30 dataset. However, NEX-DCP30 provided the following critical information at the target resolution of ~800 m: monthly average accumulation, and monthly averages of daily maximum and minimum air temperatures. As such, spatial gradients for these long-term variables in SWPar4.5 were given exactly by NEX-DCP30. Most remaining parameter mapping was informed by a combination of data from MACA and the U.S. CLIGEN ground network. To use more ground-based parameter sets than what would have been otherwise, a wider area of analysis was used with n = 485 ground parameter sets, which also addressed concerns of lacking spatial information beyond the boundary of the coverage area that is needed for geospatial interpolation. The bounding box for considered ground network locations and geospatial interpolation had corners at −121.0°W, 30.0°N, −102.0°W, and 43°N, and parameter maps were clipped to the coverage extent only after interpolation.

Prior to extracting parameter values from the ~4 km MACA dataset, preliminary steps were taken to resample the spatial resolution of MACA to the higher resolution of NEX-DCP30. First, bilinear interpolation of MACA parameter surfaces was done to produce surfaces at the target resolution. Additional to this, for grid-scale values of MEANP, SDEVP, and SKEWP, an operation was done on the bilinear interpolation surfaces to smooth the data. This addressed edge features remaining from the coarser resolution, as well as some artifacts and discontinuities apparent in long-term precipitation factors in MACA for certain locations. While the bilinear interpolation method is parameter-free, the focal point smoothing method used for the statistical distribution parameters adopted the same selected configurations for all operations. The smoothing used a circular neighborhood with a radius of seven cells around the center cell, and the median value of the neighborhood was attributed to the center cell. This neighborhood size was selected because it was comparable in scale to visual artifacts. Because of this fundamental resampling of MACA data that was the result of focal point smoothing, a quantification of the deviation from the bilinear interpolation is given in the technical validation to show the degree of change in values, assuming that bilinear interpolation did not negatively influence the performance of MACA. Focal point smoothing removed most visual artifacts that tended to resemble spatial white noise and boundary issues. In many cases, this made the degree of spatial autocorrelation of neighboring cells in affected areas closer to what would be expected.

### Delta downscaling for critical precipitation parameters

With the resampled and smoothed long-term parameter surfaces calculated from MACA, the three precipitation parameters describing the statistical moments of daily precipitation amounts, MEANP, SDEVP, and SKEWP, were involved in delta downscaling to bias-adjust grid-scale values in future time windows according to proportionality factors between equivalent grid-scale and point-scale values determined for the 40-year historical time window. To ascertain historical point-scale values, the observed ground-based parameter values were first spatially interpolated to the target resolution, as will be discussed. Adjustment factors were then given by a formulation of the delta downscaling method used in the context of long-term SWG parameters for historical and future time windows, which may be given as:

1 $${{Point}}_{f}={{Grid}}_{f}\frac{{{Point}}_{h}}{{{Grid}}_{h}}$$

where point-scale and grid-scale parameter values are denoted as being for historical (subscript h) or future (subscript f) time windows^43^. The historical window was 1974–2013 in all cases and the future windows included eight 30-year time windows at decadal intervals beginning in the year 2000. This assumed that the 40-year reference period and the 30-year future windows were equally representative of long-term factors and that overlapping time periods between the selected windows didn’t influence the bias adjustment factor. A further assumption of applying delta downscaling to future time windows is that the same proportionality factors determined for the historical period can then be used to adjust a range of grid-scale values in the future time windows. For delta downscaling in general, any percentage change in the grid-scale values is reflected in the same percentage change in the point-scale values, such that the same relative rates of change occur between the two spatial scales. In reality, non-linearity or different scaling rates in general may affect delta downscaling outcomes. However, this potential problem was mitigated by handling the data in both monthly and site-specific fashion, such that site-specific bias adjustment factors were applied to only the range of parameter value magnitudes present in a given place, month, and future time window series. Consequently, this entailed site-specific monthly bias-adjustment factors for each of the three statistical distribution parameters determined by delta downscaling.

### Parameter adjustment scheme for wet day count

After production of parameter maps for the aforementioned variables, a parameter adjustment scheme was applied to solve for the average number of days with precipitation occurrence for a given month, *n*, which is not a parameter itself but is controlled by P(W|W) and P(W|D) values. Similarly, average monthly accumulation (ACCUM) is not a parameter itself, but is found by multiplying the value of *n* by MEANP. Therefore, the parameter scheme considers known ACCUM and MEANP values and treats *n* as an unknown. As such, the scheme allows for ACCUM taken from a pre-existing dataset and estimated point-scale MEANP to both be enforced and used to then determine P(W|W) and P(W|D). To solve *n*, the scheme begins with the equation for ACCUM:

2 $$ACCUM=m\times MEANP\times \{P(W|D)/[1-P(W|W)+P(W|D)]\}$$

where the term in brackets is equivalent to *n*/*m* and *m* is the number of total days in the month. Known point-scale values of a monthly supporting variable, RATIO, were interpolated for the historical period and assumed to be unchanged over the course of climate change, defined as the ratio of P(W|W) to P(W|D). The following equations were then used to solve P(W|D) and P(W|W) by rearrangement of Eq. 1 and substitution with RATIO. First, P(W|D) may be given with the following equation:

3 $$P(W|D)=-1+\frac{1}{RATIO}+\frac{ACCUM}{m\times MEANP}$$

Then, P(W|W) may be solved as:

4 $$P(W|W)=RATIO\ast P(W|D)$$

In this scheme, the accuracy of the determined *n* value (such as displayed in Fig. 3) depends on the accuracy of the pre-determined ACCUM and MEANP values, while RATIO itself only controls the accuracy of wet/dry day sequencing for a given value of *n*. Given the limited influence of RATIO, values were not estimated for future windows, and historical RATIO surfaces were applied in all time windows. As will be discussed later, the estimation of RATIO was also associated with higher interpolation error, which may impact the sequencing of wet days.

### Empirical bayesian kriging

Empirical Bayesian Kriging (EBK) is a geostatistical interpolation technique that was involved in prediction of five parameters interpolated from observed values in the coverage area, which yielded point-scale parameter maps for the historical period^44^. These were MEANP, SDEVP, SKEWP, RATIO, and TimePk (the RATIO parameter will be discussed later). This geostatistical interpolation method was used because it addresses issues with traditional kriging methods and has automatic parameter selection, eliminating the need for calibration. EBK differs from other kriging techniques by merging multiple computational models. EBK also takes uncertainties in semi-variogram distributions into account when determining standard error by considering multiple semi-variogram models^44^. In addition to known parameter values at point locations, EBK requires co-variate raster layers of variables that have influence on the predicted parameter. For these, raster layers of monthly accumulation (NEX-DCP30), average daily maximum temperature (NEX-DCP30), and average solar radiation (MACA) were used, in addition to elevation data from 3DEP 10 m^45^. The same co-variate layers were used consistently for all five interpolated variables with varying degrees of success. The approach handled each predicted parameter and month separately, meaning that different EBK models were created for each parameter and month. Performance metrics for EBK models are given in the technical validation section.

### Gradient boosting

Gradient boosting (GB) regression implemented by ArcPro is a supervised machine learning ensemble model that uses a series of regressions to successively reduce the largest errors of previous regressions^46^. GB handles non-linearity and has fewer assumptions than multiple linear regression, though it may not always outperform multiple regression^47^. It is increasingly used in environmental applications^48^ and was applied in the present framework to estimate MX.5 P surfaces because it can additionally be used for forecasting MX.5 P for the future time windows. This is due to the fact that known values are not required once a GB model is trained, in contrast to geospatial interpolation methods. Also suiting for GB is the fact that grid-scale values of MX.5 P cannot be directly calculated from gridded climate projections of daily-scale time series (due to the inadequate temporal resolution), making delta downscaling unfeasible. A drawback of GB is that it may not result in the same degree of spatial autocorrelation as a geostatistical model because GB treats data for a single location as independent from neighboring grid cells, and therefore, GB may result in less smooth surfaces than EBK. For example, this issue may be visually evident in Fig. 4 in comparison to other surfaces. Another issue is that calibration of hyperparameters is required, though in this case, the same hyperparameters were used for all variables, leading to varying degrees of model fitting and necessitating separate cross-validations. Similar to the handling of EBK, separate GB models were trained for each calendar month, and the same co-variate map layers were used with the exception of elevation, which was omitted because of the lack of context for the use of elevation in non-geostatistical models. However, one assumption of GB is that the training data encompasses the range of input values used for predictions and forecasting over the course of climate change, which may be expected to shift in reality. This assumption is not met in all cases in the current application, such as for temperature variables.

## Technical Validation

**Fig. 6:**
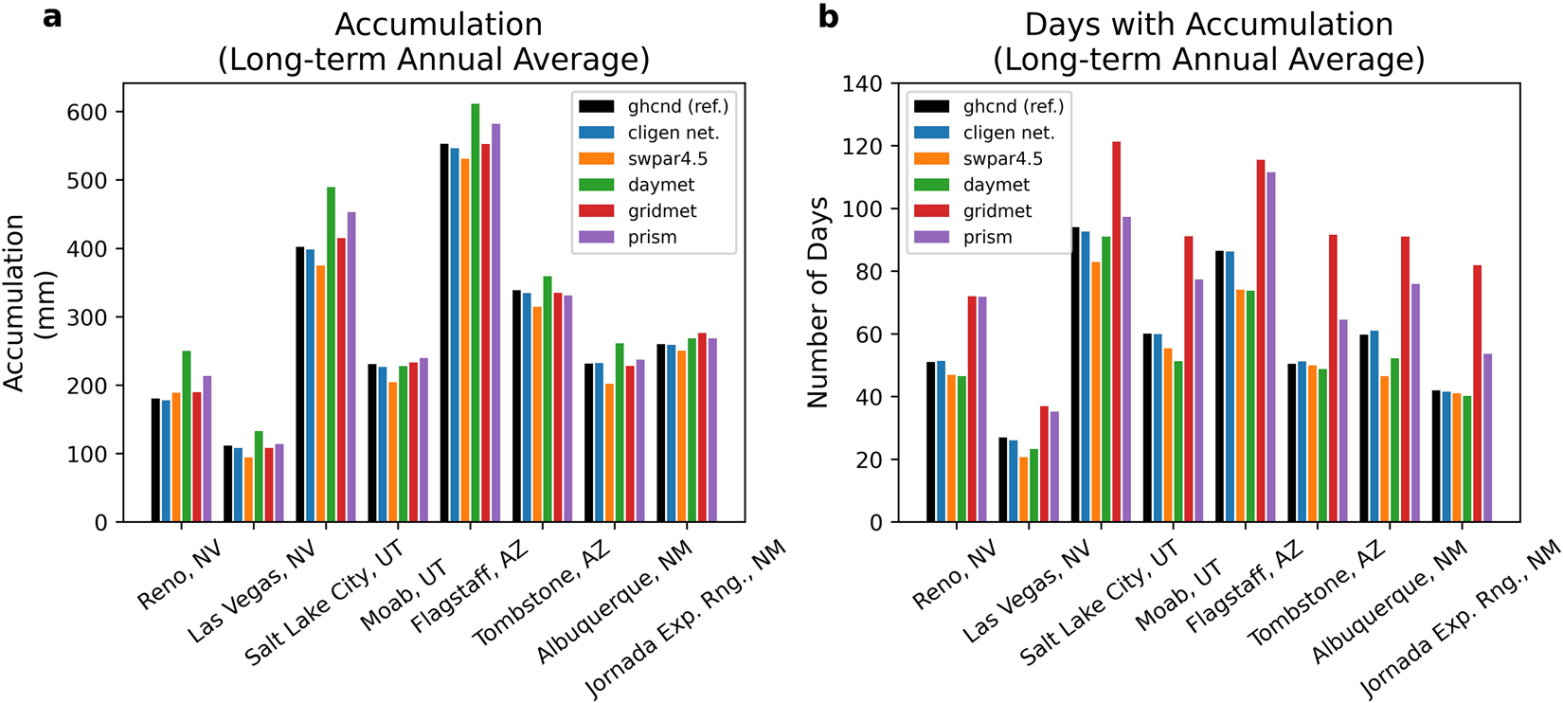
Comparison of basic precipitation factors for different datasets including (**a**) annual accumulation and (**b**) annual number of days with accumulation. The CCSM4 GCM was used to represent SWPar4.5 in this comparison for the baseline period of 1974–2013.

**Fig. 7:**
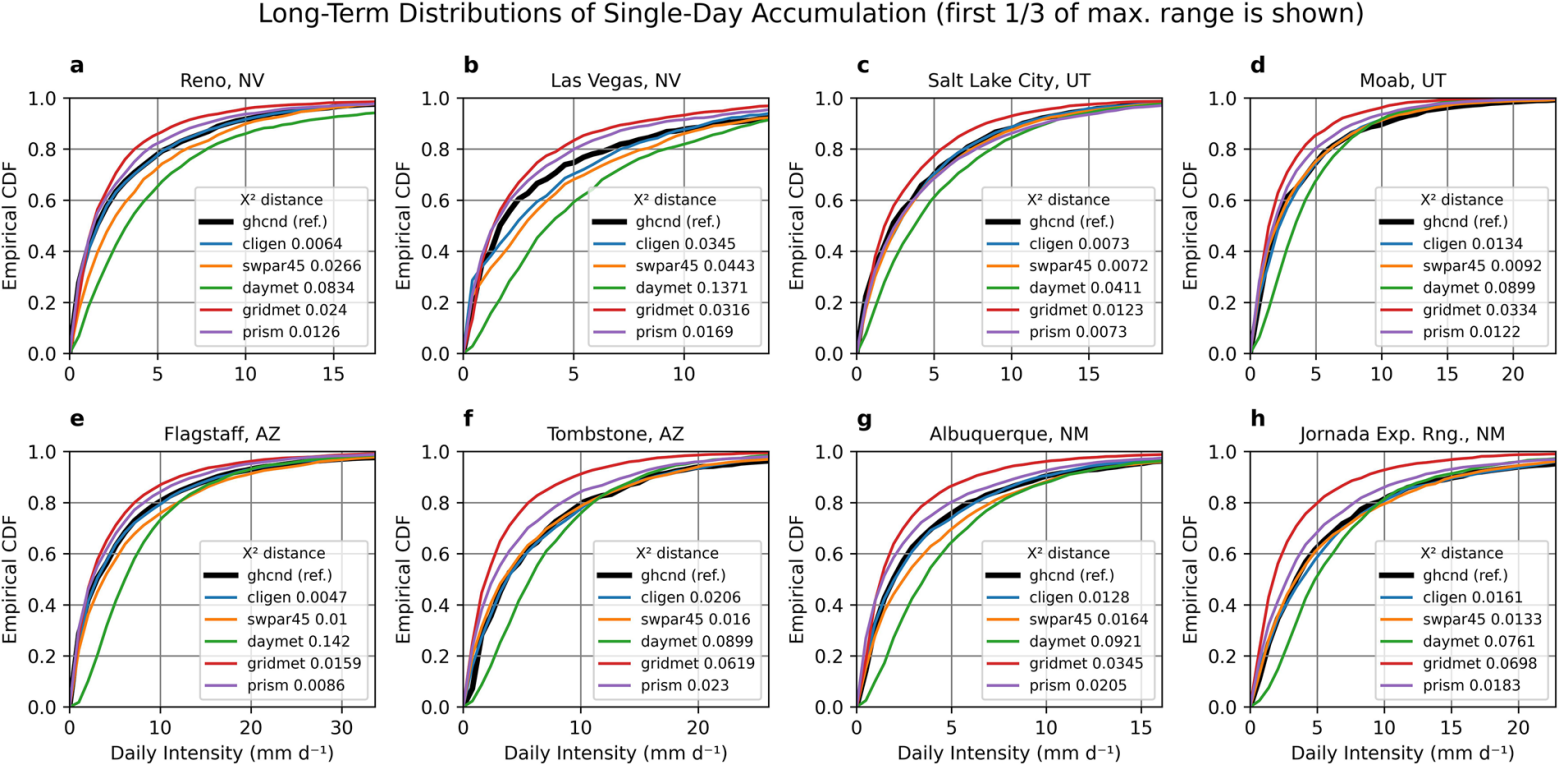
Empirical cumulative distribution functions for different precipitation datasets at the following locations: (**a**) Reno, (**b**) Las Vegas, (**c**) Salt Lake City, (**d**) Moab, (**e**) Flagstaff, (**f**) Tombstone, (**g**) Albuquerque, and (**h**) Jornada Experimental Range. The Chi-squared distance (***X***^2^) is given as a measure of deviation from the GHCNd reference curves. The CCSM4 GCM was used to represent SWPar4.5 in this comparison.

Validation was done primarily to evaluate performance of estimation methods for CLIGEN precipitation input parameters during the historical period. For this, ground-observed parameter values could be used as reference values. In the SWPar4.5 methodology, parameter estimation is represented primarily by two methods, the EBK and GB parameter mapping techniques. Additionally, the same performance metrics are shown for focal smoothing in order to quantify the difference between the bilinear interpolated surface and the focal smoothing surfaces during the MACA resampling operations. This latter step was done to reflect the deviation from raw MACA values in SWPar4.5 assuming that bilinear interpolation of MACA to the target resolution resulted in minimal change compared to focal smoothing. To a lesser degree, CLIGEN outputs were also evaluated during the historical period including validation of probability distributions of daily accumulation, which is discussed for selected locations in Figs. 6, 7. This involved the Chi-squared distance metric to quantify deviation of probability distributions from reference curves. The metric quantified the effects of parameter errors on CLIGEN output, which are compounded in the output because of propagation of error from multiple input parameters. Lastly, it was considered beyond the scope of the study to evaluate rates of change in parameter trends in historical data to extrapolate to future time windows. The performance metrics shown in the following are used to evaluate focal smoothing, EBK, and GB in Tables 3–5, respectively, and the Fig. 7 deviations^49–51^. The equation for root mean squared error (RMSE) is given as:

5 $${\rm{RMSE}}={\left(\frac{{\sum }_{i=1}^{N}{({O}_{i}-{P}_{i})}^{2}}{N}\right)}^{0.5}$$

**Table 3:** Focal point smoothing introduced some minor differences between MACA bilinear interpolation surfaces and the resulting smoothed surfaces.

Parameter	MEANP 2000…2070 1…12	SDEVP 2000…2070 1…12	SKEWP 2000…2070 1…12	MEANP 2000…2070 1…12	SDEVP 2000…2070 1…12	SKEWP 2000…2070 1…12	MEANP 2000…2070 1…12	SDEVP 2000…2070 1…12	SKEWP 2000…2070 1…12
GCM	CCSM4			CanESM2			MIROC5		
RMSE	0.0056	0.0071	0.0067	0.0058	0.0078	0.0066	0.0055	0.0071	0.0068
PBIAS	0.274	0.4364	0.3014	0.2586	0.441	0.2871	0.2792	0.4324	0.2883
MAPE	2.6787	3.7825	8.4906	2.579	3.3537	5.6816	2.6789	3.2173	5.6202

The performance metrics are averaged over the range of time windows indicated by their beginning year and over the twelve calendar months. In this case, focal smoothing is reported only for the range of time windows overlapping with future years, since EBK surfaces are used in SWPar4.5 for the historical period of 1974–2013. The unit of measurement for RMSE is inches (U.S. customary) except for SKEWP, which is unitless. The smoothed SKEWP surface showed the most change, reflecting the fact that SKEWP is likely relatively poorly predicted in the original MACA data, and in the present dataset, it is shown to be poorly predicted for the historical period.

**Table 4:** EBK performance metrics based on cross-validation.

Parameter	MEANP 1974 1…12	SDEVP 1974 1…12	SKEWP 1974 1…12	RATIO 1974 1…12	TimePk 1974 1…12	MEANP 1974 1…12	SDEVP 1974 1…12	SKEWP 1974 1…12	RATIO 1974 1…12	TimePk 1974 1…12	MEANP 1974 1…12	SDEVP 1974 1…12	SKEWP 1974 1…12	RATIO 1974 1…12	TimePk 1974 1…12
GCM	CCSM4					CanESM2					MIROC5				
RMSE	0.0374	0.0758	0.9375	1.0044	0.009	0.0372	0.0757	0.9385	1.0041	0.009	0.0373	0.0757	0.9383	1.005	0.009
PBIAS	0.1386	0.4352	0.0438	0.2039	−0.0069	0.1576	0.3836	0.0392	0.1872	−0.0071	0.1735	0.384	0.0429	0.181	−0.0068
MAPE	14.0176	15.7772	23.9339	14.4623	0.7348	13.952	15.7591	23.9536	14.463	0.7366	13.9726	15.768	23.9634	14.4666	0.7372

The unit of measurement for RMSE is U.S. customary inches, except for SKEWP, RATIO, and TimePk, which are unitless. Metrics are representative of the 1974–2013 period and are averaged by calendar month.

**Table 5:** GB performance metrics based on cross-validation.

Parameter	MX.5 P 1974 1…12	MX.5 P 1974 1…12	MX.5 P 1974 1…12
GCM	CCSM4	CanESM2	MIROC5
RMSE	0.0401	0.0344	0.0396
PBIAS	−0.114	−0.4152	−0.0807
MAPE	4.5585	3.4635	4.384

The unit of measurement for RMSE is U.S. customary inches/hr. The metrics are representative of the 1974–2013 period and are averaged by calendar month.

The following equation for percent bias (PBIAS) is given as:

6 $${PBIAS}=\frac{{\sum }_{i=1}^{N}{O}_{i}-{P}_{i}}{{\sum }_{i=1}^{N}{O}_{i}}\times 100$$

The following equation for mean absolute percentage error (MAPE) is given as:

7 $${MAPE}=\frac{1}{N}{\sum }_{i=1}^{N}\left|\frac{{O}_{i}-{P}_{i}}{{O}_{i}}\right|\times 100$$

The following equation for Chi-squared distance (*X*^2^) is given as:

8 $${{\rm X}}^{2}=\frac{1}{2}{\sum }_{i=1}^{N}\frac{{\left(O-P\right)}^{2}}{O+P}$$

where *O* and *P* are observed and predicted values, respectively, and *N* is sample size. The *X*^2^ metric is applied to the empirical PDFs corresponding to the CDF curves shown in Fig. 7 using binned data with n = 50 bins.

The evaluation of performance for EBK and GB estimation methods used separate cross-validation schemes that account for the potential for overfitted models. The EBK models are more computationally efficient to calibrate/train, allowing leave-one-out cross-validation to be done using every ground sample exactly once in the interpolated area. This differed for the GB models because retraining the model for leave-one-out cross-validation for every ground sample was less computationally efficient, and a cross-validation involving fewer permutations of the data pool was used instead. For the GB cross-validation, a K-folds approach was used with random replacement, 10 folds, and 10% of the samples being used for validation of each fold. The permutation closest to the median R-squared value was used for reporting, resulting in fewer tested samples than with leave-one-out cross-validation for the reported metrics. Similar to how EBK cross-validation was handled, separate evaluations were done for each GB model. For reporting, the 12 monthly performance metric values during the historical period for EBK and GB were averaged. Averaging was additionally done over time windows for reporting focal smoothing performance metrics.

As seen in Table 3, focal smoothing was associated with higher error for producing SKEWP parameter surfaces. This partly reflects the inherent difficulty in estimating skewness of a statistical distribution, but it also reflects a greater degree of smoothing of MACA skewness surfaces that displayed considerable noise and spatial heterogeneity, which in turn increased the degree of change from the bilinear interpolation surfaces. Both the SKEWP and RATIO parameters were also associated with higher error in the EBK cross-validation. It is important to note that compounding errors from the applied methods and individual parameters in each parameter set can potentially have a negative impact on CLIGEN outputs and derived calculations. This may partly be the cause of larger errors for some of the empirical CDFs in Fig. 7, as indicated by the chi-squared distance metric. Figure 7 also shows that SWPar4.5 has less consistency in the position of SWPar4.5 curves relative to the reference curves compared to other datasets. Looking at some locations, an underestimation bias may be apparent, though SWPar4.5 provided excellent approximations of ground distributions in other cases. There may also be an issue with model overfitting, such as in the Las Vegas analysis where the CLIGEN network disagreed with the GHCNd reference distribution. Therefore, further effort is needed to validate CLIGEN outputs and derived factors, which may be necessarily carried out for individual output factors separately, as behavior changes from factor to factor. Improvements to this framework are likely possible as updated datasets and more refined models become available. Overall, estimation errors were low, indicating that the framework has the potential to be a valuable tool for climate impact assessment.

### Comparison of precipitation datasets to SWPar4.5

The ability of CLIGEN time series to reproduce basic precipitation factors in comparison to other datasets is shown in Figs. 6, 7 for the historical period of 1974–2013. The comparison includes CLIGEN time series generated from observed parameter sets from stations in the U.S. CLIGEN ground network. Also included are time series based on SWPar4.5 using the Community Climate System Model 4.0 (CCSM4) GCM. This GCM is recommended here as the most “typical” GCM of the three selected GCMs in SWPar4.5. The CCSM4 model was found to be representative of the CMIP5 ensemble precipitation outcomes and ranked high in prediction of average annual precipitation, while the two other GCMs provided contrasting precipitation outcomes. The considered ground locations that are shown had observations used as references with high record completeness from the NOAA-GHCNd ground network^52^. Grid-scale daily datasets commonly used for environmental assessment are also shown, being PRISM daily (~4 km), GridMet (~4 km), and DayMet (~1 km). The reference periods that were queried to calculate long-term baselines varied in availability, as indicated in Table 1, with differing starting years for each dataset.

Basic precipitation factors in Fig. 6 compare long-term average annual accumulation and the number of days with precipitation. When considered together, these two factors serve as a check for whether the mean daily intensity of respective datasets is realistic. Similarly, in Fig. 7, empirical cumulative frequency distributions of daily non-zero accumulation present in time series data shows differences in daily intensity for the bottom one-third of the distribution at each site. The chi-squared distance metric is shown in Fig. 7, which quantifies deviation of each estimated probability distribution from the reference distribution. Considering all factors, differences between point-scale and grid-scale datasets are apparent in this comparison, as are differences that arise due to downscaling methodology. The drizzle effect may also be apparent in Figs. 6, 7 looking at PRISM, and more so, the GridMet distributions. Both consistently show a greater number of days with precipitation than the ground record, and their CDF distributions rise at a faster rate than the reference distributions, reflecting a greater proportion of events with small accumulation amounts. Contrastingly, DayMet factors may actually be indicative of greater intensity than the other data products by having a CDF that rises at a slower rate than the reference data, unlike the other gridded datasets. The ~4 km MACA dataset that was applied in the present framework uses a similar downscaling method as GridMet, resulting in their precipitation frequency distributions being likewise similar.

Ranking datasets by their ability to estimate factors at the sites in Figs. 6, 7 indicates that SWPar4.5 and PRISM achieve similar prediction accuracy for both long-term annual accumulation and CDFs of daily accumulation, while SWPar4.5 is considerably better at predicting the number of wet days compared to PRISM. Using percent error (PE) and chi-squared distance as performance measures averaged across the eight sites, the PE for long-term accumulation was 6% and 8% for PRISM and SWPar4.5, respectively, while GridMet performed the best with 3% PE. The relative distances between CDFs showed that site-averaged chi-squared distance for SWPar4.5 and PRISM were roughly equal (0.018 and 0.015, respectively), while GridMet and DayMet had substantially larger chi-squared distances. In terms of the number of wet days, site-averaged PE of SWPar4.5 was reasonably estimated at 11% compared to 27% for PRISM, while GridMet had 53%, and DayMet had 9%, indicating large disagreement between the gridded datasets. Therefore, results show that SWPar4.5 is able to achieve an overall balance of predicting daily accumulation amounts while maintaining accuracy in both net accumulation and the number of wet days.

### Comparison of SWPar4.5 regional ensemble statistics

**Fig. 8:**
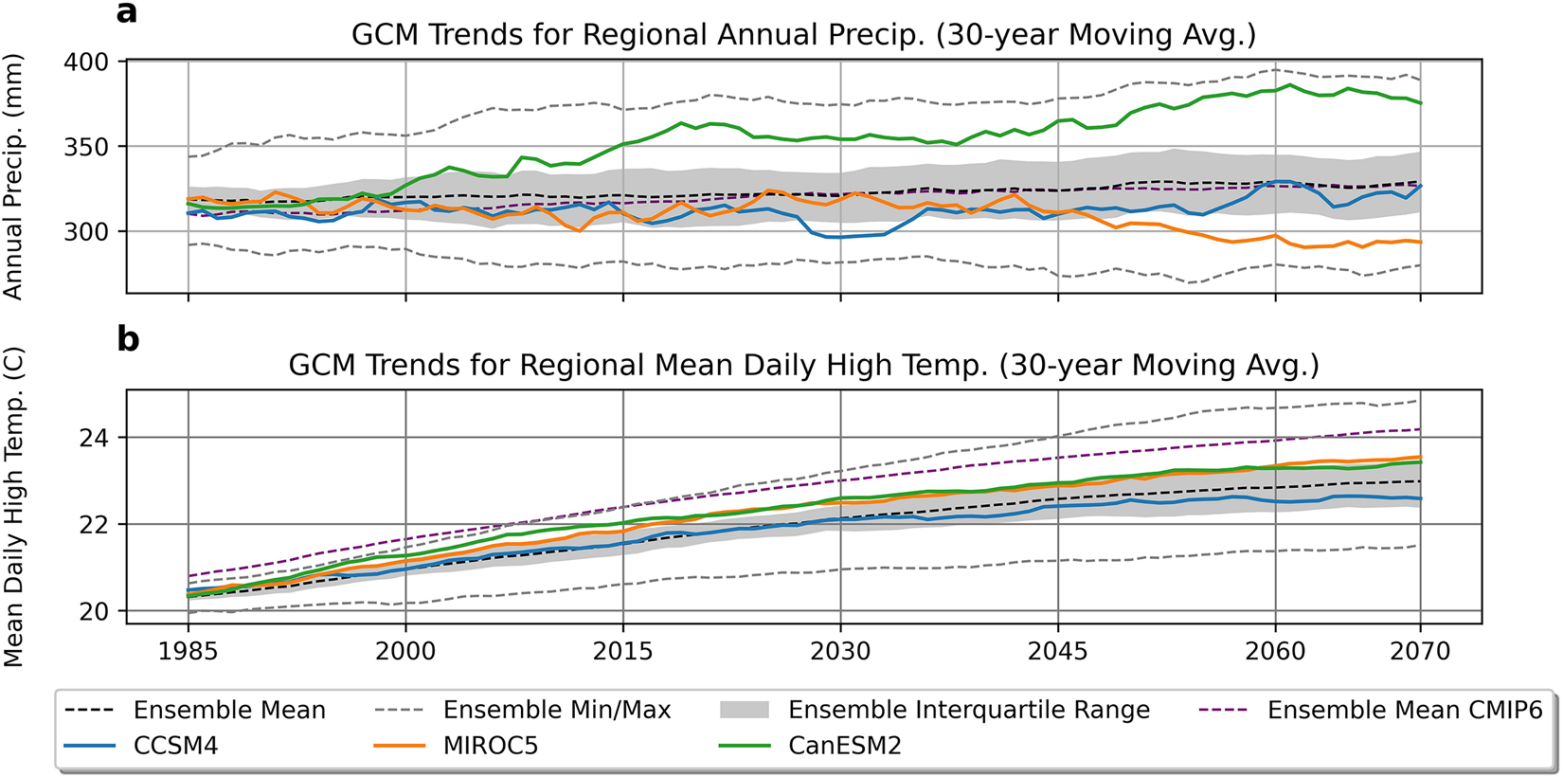
Regionally averaged ensemble statistic trends and individual GCM trends for (**a**) 30-year average accumulation and (**b**) 30-year average mean daily high temperature. The ensemble statistics are derived from the CMIP5 ensemble, except for the ensemble means of CMIP6 being additionally shown for comparison purposes.

Regional analysis of the CMIP ensembles in Fig. 8 shows the spatially averaged precipitation and temperature trends that were considered in the selection of the three GCMs in SWPar4.5. The CMIP5 ensemble statistics in Fig. 8 are derived from 21 available GCMs in the NEX-DCP30 product. For comparison purposes, the ensemble mean of the newer CMIP6 trends were calculated from a coarser product (NEX-GDDP^53^) with 30 available GCMs. It can be seen that change in cumulative precipitation is relatively small, and greater variance in the ensemble occurs compared to temperature. The comparisons indicate that CMIP5 and CMIP6 have the same general outlook. However, CMIP6 predicts a baseline with hotter temperatures, and subsequently, hotter temperatures at the end of the century, increasing 2–3 °C overall (~2.7 °C). This represents an important difference of CMIP5 and SWPar4.5, because historical baselines were considerably different in the updated CMIP6 ensemble. Further investigation is needed to understand the impact of how temperature-driven factors may differ between the two ensembles. Obvious processes such as evapotranspiration, snowmelt, and ecosystem function are likely to be affected in addition to the apparent temperature-driven precipitation changes that have been discussed.

Also shown in Fig. 8 are trends for the three different GCMs selected to be represented in SWPar4.5, being CCSM4, CanESM2, and MIROC5. The three GCMs were selected to provide contrasting precipitation outcomes because there is larger variance in the ensemble precipitation trends in comparison to temperature. We recommend CCSM4 for any analysis that considers only one set of climate inputs because CCSM4 closely follows the ensemble means of accumulation and temperature and was found to have a high RMSE performance score in predicting historical baselines for annual precipitation (compared to other GCMs in the ensemble by having the 2^nd^ lowest overall RMSE for average annual precipitation). Much of the outputs of SWPar4.5 demonstrated in the present paper use CCSM4 for this reason. The other two additional GCMs, CanESM2 and MIROC5, provide high- and low-end precipitation outcomes, respectively, and both predict hotter conditions by the end of the century, more similar to temperatures of CMIP6. For applications like water erosion modeling, the range of precipitation trends given by the three GCMs allows for uncertainties of future erosion rates to be derived.

## Usage Notes

Tools were created to assist with querying the geodatabases for a given location and time window to then produce parameter files with the particular text formatting that is necessary for use with CLIGEN. The easiest option for doing this is to access the following link to download individual parameter files: https://apps.tucson.ars.ag.gov/cligenpar. For batch queries, a Windows OS command line executable was created that can be downloaded along with the GDBs from the previously linked data repository. Both tools apply necessary unit conversions and range checks. In most cases, U.S. customary units are used, though some variables in CLIGEN are reported in metric units, particularly in its output. This creates a mixture of U.S. and metric units that the user should take care to avoid confusing. These tools also enforce value ranges to avoid negative values and other potential non-physical values. The code base for the website and command line tool is given in the following link: https://github.com/ARS-SWRC/GDB-to-Pars-CLIGEN with CC0 license. Detailed instructions for using the command line executable are included in the link. Lastly, another option for batch querying is for the user to run a stand-alone script in Python (also available at the link), which doesn’t require an executable, but requires the stated Python libraries.

## Data Availability

The SWPar4.5 dataset^54^ is available at the ReData Repository (University of Arizona) with CC BY 4.0 license at 10.25422/azu.data.28507262. The SWPar4.5 dataset is formatted as map layers in three separate ESRI geodatabases, one for each selected GCM, totaling ~50 GB memory. The geodatabases (GDBs) may be viewed with ArcGIS or by accessing them programmatically using the GDAL library, which is currently being distributed as part of the ArcPy package. If needed, GDAL may be installed independently of ArcPy with major programming languages. The dataset represents SWG parameters covering the range from 1974–2099 for the middle ground CMIP5 RCP4.5 scenario and three selected GCMS, being CCSM4, CanESM2, MIROC5. The dataset includes all variables required to parameterize the SWG called CLIGEN except for wind variables, as outlined in Table 2. The Usage Notes section has important guidance for creating a formatted CLIGEN parameter file and obtaining appropriate wind parameter values.

## Code availability

The code base that was used for processing, analyzing, and visualizing the content of this work is available at https://github.com/afullhart/climatechange.
